# The Origins of Time-Delay in Template Biopolymerization Processes

**DOI:** 10.1371/journal.pcbi.1000726

**Published:** 2010-04-01

**Authors:** Luis Mier-y-Terán-Romero, Mary Silber, Vassily Hatzimanikatis

**Affiliations:** 1 Department of Physics and Astronomy, Northwestern University, Evanston, Illinois, United States of America; 2 Laboratory of Computational Systems Biotechnology, Ecole Polytechnique Fédérale de Lausanne, Lausanne, Switzerland; 3 Swiss Institute of Bioinformatics, Lausanne, Switzerland; 4 Engineering Sciences and Applied Mathematics, Northwestern University, Evanston, Illinois, United States of America; 5 Northwestern Institute on Complex Systems, Northwestern University, Evanston, Illinois, United States of Amerca; University of Virginia, United States of America

## Abstract

Time-delays are common in many physical and biological systems and they give rise to complex dynamic phenomena. The elementary processes involved in template biopolymerization, such as mRNA and protein synthesis, introduce significant time delays. However, there is not currently a systematic mapping between the individual mechanistic parameters and the time delays in these networks. We present here the development of mathematical, time-delay models for protein translation, based on PDE models, which in turn are derived through systematic approximations of first-principles mechanistic models. Theoretical analysis suggests that the key features that determine the time-delays and the agreement between the time-delay and the mechanistic models are ribosome density and distribution, i.e., the number of ribosomes on the mRNA chain relative to their maximum and their distribution along the mRNA chain. Based on analytical considerations and on computational studies, we show that the steady-state and dynamic responses of the time-delay models are in excellent agreement with the detailed mechanistic models, under physiological conditions that correspond to uniform ribosome distribution and for ribosome density up to 70%. The methodology presented here can be used for the development of reduced time-delay models of mRNA synthesis and large genetic networks. The good agreement between the time-delay and the mechanistic models will allow us to use the reduced model and advanced computational methods from nonlinear dynamics in order to perform studies that are not practical using the large-scale mechanistic models.

## Author Summary

Genetic networks display exceedingly complex and rich behavior which is modulated by multiple mechanisms, including many diverse types of interactions between DNA, mRNA and protein molecules. Mathematical models of gene networks must necessarily consider the essential mechanistic details of the processes involved in order to make reliable predictions. However, even though the description of the process becomes more accurate as more mechanistic details are incorporated into the mathematical model, the added mathematical complexity will make it difficult to parameterize and extract information from such models given the limited amount of experimental data. Protein synthesis is precisely one of the phases in the network machinery where certain mechanistic details are important and should thus be taken into account. Here, we develop a methodology to reduce a mathematical model for protein synthesis by performing approximations on a mechanistic model, retaining the essential details of the process. Our methodology opens up the possibility of utilizing powerful mathematical tools, such as bifurcation analysis, for understanding the complex dynamics displayed by genetic networks and design strategies for metabolic engineering and synthetic biology.

## Introduction

### Time-Delays in Mathematical Biology

Time-delay models are common in mathematical biology, as is demonstrated by the use of mathematical models incorporating time-delays in a wide range of applications. These include population dynamics, the chemostat, neural networks, blood cell maturation, transcriptional regulator dynamics, virus dynamics and genetic networks [1]–[9]. In the context of protein synthesis in genetic circuits, time-delay arises from the series of steps required between the expression of individual genes to the production of the corresponding protein. The main processes that contribute to the time delay are promoter induction, mRNA transcription, transport, splicing and processing, as well as protein translation.

Complex dynamical behavior can arise as a consequence of time-delays in a system. Biological systems with significant time delays may exhibit limit cycle oscillations and chaos [10]. In addition, incorporating time delays in models of gene networks is often essential to capture the whole range of dynamic behavior. For example, a single self-repressed gene has been observed in experiments to display oscillatory behavior which cannot be captured by models that ignore the time delay required to obtain a finished protein from the expressed gene. However, this oscillatory behavior is reproduced by a mathematical model in terms of time delayed differential equations [7]–[9]. In addition, mathematical analysis that ignored time-delays led to the erroneous conclusion that oscillations were not possible for this single gene, and this conclusion led to a potentially misleading hypothesis [11].

Gene regulatory circuits possess incredibly diverse functions. They function as molecular switches, molecular clocks or as sensors which are able to discriminate noise in the input [12]–[14]. One goal of synthetic biology and metabolic engineering is the design of synthetic networks with desired circuit functionalities [15],[16]. The efficient design of these circuits requires guidelines obtained from mathematical models that account for the essential mechanistic details of the system through a systematic framework. The time delays associated with mRNA and protein expression are usually significant for the complex dynamics of gene regulatory circuits and must therefore be incorporated into the mathematical models in a systematic fashion.

### Background

**Figure 1:**
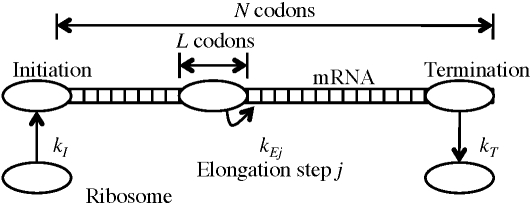
Schematic view of the translation process.

We focus on the mathematical modeling of protein synthesis (translation), which is central to cellular processes and gene networks. Translation is divided conceptually into three stages: initiation, elongation and termination (Figure 1). First, the ribosomal subunits bind at the initiation site of the mRNA and assemble the ribosome (initiation). Then, in a repetitive manner, the ribosome adds one amino acid to the partial polypeptide chain and it translocates one codon forward (elongation). Finally, the ribosome reaches the stop codon and detaches from the template releasing the completed protein molecule (termination). Many molecular components are required for translation, all working in conjunction. Furthermore, several ribosomes go through the elongation process simultaneously on the same mRNA chain, forming a structure called polysome, or polyribosome, which can be visualized [17], and can be quantified using biochemical and biophysical methods [18],[19]. Polysome size refers to the number of ribosomes bound to a particular mRNA at one time. The protein translation components comprise around half the dry weight of the cell and up to 80% of its energy.

Mathematical models of protein synthesis have used many diverse approaches and incorporate different levels of mechanistic detail. In [20], the authors developed a deterministic Markov model for RNA transcription and protein translation. In this model, the DNA and mRNA templates can only be bound with either a single RNA polymerase or a single ribosome, respectively. They obtain a compartmental model in which DNA and mRNA templates flow between compartments as the degree of transcription or translation advances without explicitly accounting for the dynamics of RNA polymerases and ribosomes. The work of MacDonald et al., [21],[22], was one of the first instances in which the dynamics of ribosomes on the mRNA was explicitly considered. The model is written in terms of deterministic rate equations for the ribosomal fluxes on the mRNA templates and captures ribosome sequestration on the chains. The studies of several authors and the model extension by Heinrich and Rapoport has provided a good understanding of the effect of ribosome dynamics in protein translation [23]–[25]. More recent studies have concentrated on performing genome-scale analysis of expression levels and on including the effect of the sequence of reactions that occur at each elongation step [26]–[29].

Stochastic effects in genetic circuits and protein translation have also been considered [30]–[37]. Efficient algorithms and software packages exist for the stochastic modeling of large-scale chemical systems, and in particular, for gene regulatory networks [38]–[44]. Ribosome elongation has also been modeled as a driven gas in a one-dimensional lattice with hard-core repulsion, the so-called totally asymmetric exclusion process (TASEP) with stochastic dynamics [45]–[50]. The TASEP model has demonstrated that the translation system is capable of undergoing both first and second order phase transitions, exhibiting jumps in the ribosomal density and current [51],[52]. It is possible to view the protein translation models of [21]–[23] as a TASEP with deterministic dynamics in terms of ODEs with a mean field approximation. Other studies have concentrated on developing reduced stochastic models of gene regulatory networks. One possible reduction approach is to lump chemical processes such as transcription and translation and model them via time-delays [53]–[56]. This model reduction has shown that stochastic effects in a gene regulatory network may induce behaviors not captured by a deterministic formulation [53].

A detailed consideration of ribosome dynamics in protein translation usually leads to models with large numbers of differential equations, complicating the mathematical analysis. For this reason, when modeling genetic circuits mathematically, it is common practice to use heuristic arguments and consider protein synthesis as proportional to the amount of mRNA present, on occasion including a time delay [5]–[9],[15],[16]. However, it is well known that mRNA and protein levels do not display an exact correlation [57]–[60], and that the complex coupling of ribosome dynamics with protein synthesis is at least partly responsible for this. The rate of protein synthesis is related to the ribosome loading of its mRNA, though this loading shows high variability across mRNA species [61]. Moreover, it has been shown in experiments that the concentration of free ribosomes is limiting for protein synthesis in *E. coli* [62], and computational studies suggest that the translation machinery is very sensitive to this concentration [27], as well as to the kinetic parameters of the translation process. Well established experimental techniques are able to measure translation rates and monitor time courses of protein levels, [63]–[65], and these techniques allow the observation and quantification of non-negligible translational time delays [64]. All this evidence suggests that the commonly used modeling framework of taking the protein synthesis rate to be proportional to the delayed concentration of mRNA, while useful to obtain some information on the behavior of genetic circuits, is not suitable in situations where ribosome dynamics are known to be of importance. This motivates the use of more detailed modeling that accounts for the mechanistic details of the elementary steps of translation.

### Objectives of Present Study

We present here a systematic mathematical framework for the development of a delay differential equation model of template polymerization, such as mRNA and protein synthesis. Our focus has been primarily on protein translation, which represents the main source of delay in bacteria where transcription and translation occur simultaneously. The framework is based on systematically approximating a mechanistic mathematical model of ordinary differential equations (ODEs), first derived in [21]–[23], by a continuum model in the form of a partial differential equation (PDE) model and showing rigorously that this PDE model is completely equivalent to a time delay model. This time-delay model is a generalization of the model proposed heuristically in [23].

The delay model derived here offers many advantages. First, the systematic framework guarantees that all relevant aspects of the mechanistic model are preserved in the approximation, and it allows a systematic investigation of the validity of the approximations. Second, our reduction of the mechanistic model provides a powerful conceptual picture in which the essential aspects of protein translation are preserved and the numerous mechanistic parameters are condensed into the essential parameters of the process. Third, the time-delay model circumvents the impracticality of the large number of ODEs of the mechanistic model and the framework developed here is amenable to well known computational tools for bifurcation analysis of delay differential equations [66] (manuscript in preparation). This type of analysis allows us to efficiently explore the system's behavior in extensive regions of parameter space. Fourth, the delay model is easily parametrized and the rigorous map between parameters of the time-delay and mechanistic models may be complemented by using well known experimental methods for obtaining the time delay resulting from protein translation [63],[64].

## Methods

### Background: Mechanistic Model of Protein Synthesis

The mechanistic model for protein translation of Heinrich and Rapoport [23], takes into account the sequestration and dynamics of ribosomes on mRNA templates. These are essential aspects of the process as it has been shown experimentally and computationally that free ribosomes limit protein synthesis in *E. coli* [27],[62]. The model has been shown to capture qualitative and quantitative aspects of the process, such as steady state and dynamic agreement, as well as the ribosomal distribution along the mRNA molecule [23],[25],[31]. Commonly used models of protein translation cannot capture these aspects because they do not describe the detailed translation process.

The model described in this section is a version of a TASEP model with deterministic dynamics in terms of ODEs, where a mean field assumption was made. In contrast with typical TASEP models, which consider either constant initiation rates or periodic boundary conditions, the present model considers a finite pool of ribosomes and their initiation rate is a function of the free ribosomes.

The model of [21]–[23] has the form of an ODE system and considers 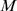 identical mRNA molecules, each with 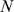 codons, and 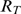 total ribosomes, both per unit volume. Ribosomes are modeled as hard bodies that cover 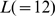 codons on the mRNA chain. The variables of the system are the probabilities that each codon 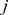 is occupied by the front of a ribosome and denoted by 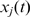, 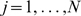. Explicitly, we look at all copies of a single-species mRNA and take 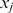 as equal to the total number of ribosome fronts on codon 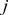 on all mRNA copies, divided by the total number of copies.

Treating variables as continuous functions of time, the system dynamics is described by the following system of ODEs
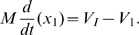
(1a)

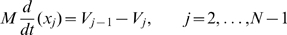
(1b)

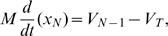
(1c)where 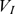 is the initiation rate, 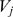, for 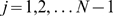, the elongation rates, 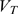 the termination rate and 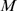 the concentration of mRNAs.

The initiation rate, 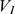, is given by
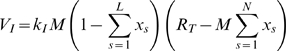
(2)and it is proportional to the number of mRNA molecules with a free initiation site, 
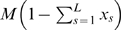
, and to the number of free ribosomes, 
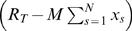
, whose number decreases due to ribosomes occupying the template. We denote the initiation rate constant by 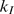.

The elongation and termination fluxes, 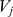 and 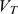, respectively, are given as
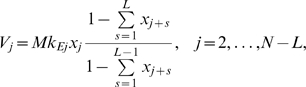
(3a)

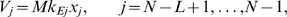
(3b)

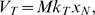
(3c)where 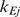 and 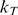 are the elongation and termination rates constants, respectively, and the fraction in Eq. 3a approximates to the conditional probability that codon 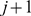 is empty given that 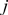 is full and it accounts for steric hindrance. This factor is absent from Eqs. 3b and 3c since ribosomes unbind once their fronts reach codon 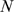 and therefore there is no hindrance effect for the last 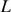 codons.

The reactions in the model are assumed to be irreversible and the reversible association of ribosomes from its subunits is not explicitly modeled. Dilution of concentrations due to cell growth is not included in the model. In general, the reaction rate constants 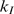, 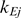, 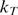 and total ribosome concentration may be time-dependent as the additional chemical components involved in initiation, elongation and termination may vary in time. For *E. coli*, typical ranges for the kinetic parameters and other relevant quantities in the model are given in Table 1.

**Table 1:** Typical translation parameters for *E. coli*.

Notation	Description	Typical Value	References
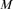	mRNA concentration	1400 molecules/cell volume	[67],[68]
–	Single mRNA species copy number	10–100 molecules/cell volume	[68]
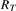	Total ribosome concentration	7,000–70,000 molecules/cell volume	[67],[68]
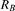	Bound ribosome concentration	0.8 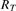	[68]
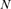	mRNA size	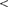 100–1700 codons	[92]
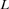	Ribosome length	12	[67],[93]
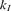	Initiation rate	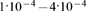 cell volume/sec^a^	–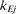	Elongation rate at codon 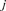	10–20 amino acids/sec	[67],[68]
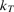	Termination rate	10–20 amino acids/sec^a^	–
–	Time between initiation events	3.2 sec	[69]
–	Space between translating ribosomes	40–80 codons	[68]
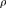	Density	0.15–0.3^b^	–

*^a^*Value chosen to yield uniform distribution of ribosomes [27]. Experimental observations show that initiation is the rate limiting step of translation [18] and [19]. This yields steady state ribosome distributions with low and nearly uniform amplitude along the mRNA chain [23].

*^b^*Inferred from the ribosome length, 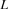, and the typical ribosome spacing on the mRNA template.

The protein production rate is equal to the rate of ribosomes terminating at the last codon and it is described by the following equation
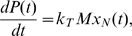
(4)where 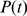 is the protein concentration.

Some additional fundamental quantities in the mechanistic model are the concentration of bound ribosomes
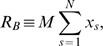
(5)and the related ribosome density, defined mathematically as
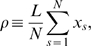
(6)which represents the fraction of the mRNA covered by ribosomes. In terms of the bound ribosome concentration, the mean polysome size is simply 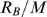.

## Results

### Parameter and Variable Non-dimensionalization

We first introduce new dimensionless variables and parameters for the system, using characteristic values for the total ribosome concentration, 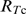, the elongation rate constant, 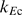 and the mRNA codon number, 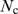, (Table 2). We define:

- The dimensionless mRNA, ribosome and protein concentrations:
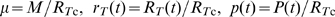
(7)through the scaling by the characteristic value for the total ribosome concentration, 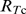.
- The dimensionless time variable:
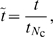
(8)scaled by the time it takes to synthesize a completed protein, 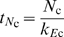. A ribosome located at codon 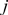 with codon 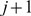 empty will elongate in a time 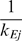, where 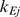 is the elongation rate constant. The total elongation time for an mRNA of 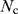 codons is therefore 
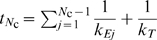
. The scaling chosen follows after considering 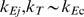, 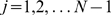. Henceforth, the tilde in the non-dimensional time variable is omitted for notational convenience.
- The dimensionless, time-dependent, rate constants of initiation, elongation and termination:
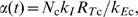
(9a)

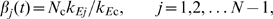
(9b)

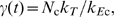
(9c)respectively. The first of these includes an additional factor of 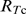 since it deals with a bimolecular reaction.

**Table 2:** Non-dimensional quantities introduced.

Notation	Definition^a^	Description of non-dimensional quantity
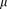	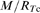	mRNA concentration
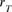	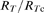	Total ribosome concentration
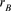	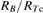	Bound ribosome concentration
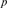	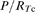	Protein concentration
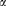	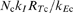	Initiation rate
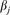	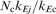	Elongation rate
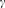	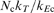	Termination rate
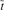	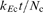	Scaled time

*^a^*The quantities 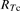, 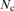 and 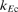 represent characteristic values of the total ribosome concentration, the mRNA codon number and the elongation and termination rate constants, respectively.

The mechanistic model, Eqs. 1, may now be written in non-dimensional form:
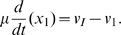
(10a)

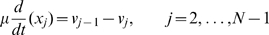
(10b)

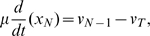
(10c)where 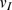, 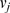, for 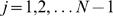 and 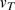 are the non-dimensional initiation, elongation and termination rates. They are given by
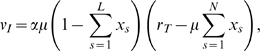
(11a)

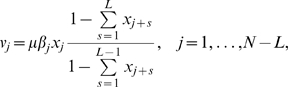
(11b)

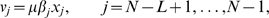
(11c)

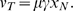
(11d)Finally, we have the non-dimensional versions of the bound ribosome concentration, Eq. 5, and of the ribosome density, Eq. 6,
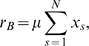
(12a)

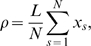
(12b)respectively.

### Physiological Conditions: Translation is Initiation Limited

Experimental data suggests that for most mRNAs in many organisms, the rate limiting step of the translation process is initiation [18],[19],[67],[68]. This aspect of the translation process leads to a steady state distribution in which ribosomes are uniformly distributed along the mRNA chain with minimal impact on steric hindrance. Ribosomal densities have been measured experimentally for both prokaryotes and eukaryotes: in *E. coli* the ribosomal density averaged over all mRNA species is around 0.3 [67],[69], and 0.2 in *S. cerevisiae* [18]. Generally, prokaryotes tend to have larger ribosomal densities than eukaryotes [67]. Simulations of Eqs. 1 under such initiation limited conditions result in steady state ribosome distributions with small density that are nearly uniform along the mRNA chain.

In general, the number of total ribosomes can vary due to changes in the synthesis of their components. In addition, changes in the components of the initiation, elongation and termination processes lead also to changes in the values of the corresponding rate constants. The primary cause responsible for the changes in these rate constants are changes in the availability of amino acids. If we assume that the time average values, 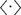, of total ribosomes and elongation rate constants are equal to their characteristic values
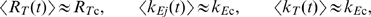
(13)we obtain
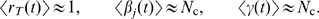
(14)The value of the initiation rate constant can vary independently and determines the ribosome density. A simple estimate shows that, in order to achieve a uniform ribosome distribution with low amplitude, the initiation rate constant must also vary slowly around some mean value and be sufficiently small (Text S1):
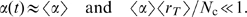
(15)


### PDE Formulation

The low density, slowly varying ribosome distribution conditions occurring in the initiation limited regime permit the use of two approximations: *(i)* a mean field ribosome distribution to effectively approximate steric hindrance effects (Text S2) and *(ii)* a hydrodynamic approximation to obtain a PDE model as a reduction of the mechanistic one (Text S3).

The reformulation of the mechanistic model via the hydrodynamic approximation is based on the following principles:

- The discrete description of the codons, labelled as 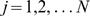, is replaced by a continuous variable 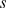 which measures length along the chain, such that 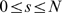. In this reformulation, codon 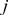 corresponds to the segment 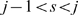 of the complete domain 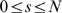.
- The number of ribosomes per codon, which was represented by the variable 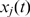 as in Eqs. 10, is described by their number per unit length of the chain, 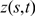, so that 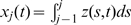. It is assumed that the ribosome distribution, 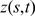, is not far from the uniform one.
- The elongation rate constants, 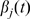, are assumed to be slowly varying as a function of the codon index 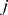 and are extended along the domain 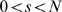 to a continuous, slowly varying version denoted as 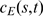 and referred to as the *velocity function*, as it quantifies the rate of progression of ribosomes along the template. Its use is equivalent to assuming that ribosomes move along the mRNAs continuously in space instead of advancing discretely one codon at a time.

The dimensionless PDE model which approximates the mechanistic model of Eqs. 10 is described by the following equations
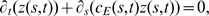
(16a)

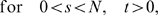


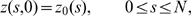
(16b)

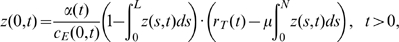
(16c)The function 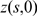 is the initial distribution of ribosomes on the mRNAs at time 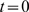 and it is denoted by 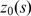. The function 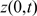 is the ribosomal density at the 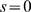 boundary and it is determined by the boundary condition described in Eq. 16.

Hydrodynamic approximations have been used in the past in the context of models of TASEP and have yielded non-linear diffusion PDEs for such processes [47], [70]–[75]. Here we retain only the most dominant terms, i.e., the convective ones (Text S3), in order to derive a reduced time-delay model based on the mechanistic description.

In the special case when the elongation rates constants are time-independent and vary only from codon to codon [28], [76]–[79], the velocity function depends only on the space variable 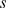. Under initiation limited conditions, numerical experiments from [23] show that the ribosomal distribution attains a steady state essentially after one elongation period. In the case of eukaryotes, where mRNA chains may have half-lives of several hours, most of the protein synthesis carried out occurs under steady state conditions. Because of this, steady state solutions are commonly considered in translation modeling [21]–[23],[31]. The present PDE formulation of translation may be used to show that in this special case of elongation rate constants that only vary from codon to codon, deviations in the ribosome distribution from the steady state decay in time, i.e. the steady state ribosome distribution is stable (Text S5).

The following system properties can then be formulated in terms of the PDE model variables:

- The concentration of bound ribosomes (Eq. 12a)
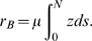
(17a)
- The ribosome density (Eq. 12b)
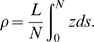
(17b)
- The initiation rate is given by (Eq. 11a)
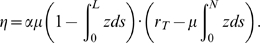
(17c)
- The non-dimensional rate of protein production is (Eq. 4)
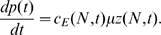
(17d)

### Delay Model

We used the PDE formulation of the problem to derive a time-delay model of protein synthesis (Text S3). The delay model is first expressed as an integral equation for the initiation rate. For time-independent kinetic parameters it takes the form
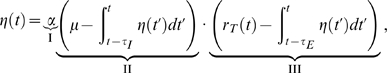
(18)where 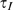 and 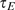 are the initiation and elongation time-delays, respectively, and are given explicitly below. Equation 18 is complemented by a history function 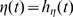 for 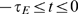. The three terms in Eq. 18 arise from mass action kinetics for a bimolecular reaction and correspond to: the initiation rate constant (I), the concentration of mRNAs with a free initiation site (II) and the concentration of free ribosomes (III).

The time delay appears in our model through two mechanisms, the initiation time delay
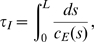
(19a)which is equal to the traversal time of the first 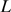 codons, and the elongation time delay
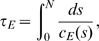
(19b)which is equal to the traversal times of the complete 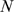 codons. We give more general expressions for the delays in Text S3 (Eqs. S3.17 and S3.19) in the case of time-dependent rate constants.

The integral equation is complemented by the description of protein production:
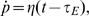
(20)obtained from Eq. 17d and it captures the fact that, in this new formulation, proteins produced at time 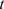 are the result of ribosomes that initiated at time 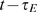.

We use the simple transformation 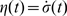 to recast Eq. 18 into a delay differential equation for 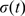, and the final delay model is
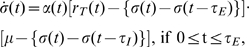
(21a)

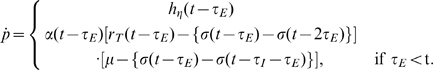
(21b)We note from Eq. 20 that protein production in the interval 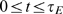 is given by the history function of the initiation rate, 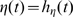, for 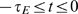.

Our systematic reformulation of the problem into a delay differential equation offers two main advantages. First is the lumping of 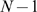 elongation and termination rate constants, 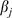
's and 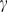, into two parameters, the initiation and elongation time delays 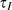 and 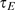. One may use known experimental techniques to parameterize the delay model and obtain the delay times of Eqs. 19 [63],[64].

Second, modeling the problem with a single delay equation facilitates the use of numerous analytical and computational tools for the analysis of equations of this type, such as bifurcation analysis [66] (manuscript in preparation). Using these tools we can explore the dynamic behavior of the system in wide regions of parameter space and the properties of genetic networks, such as the repressilator [16]. This type of parameter exploration would prove difficult if the system were modeled by a large number of ODEs.

Our model reduction is similar in spirit to the work in [20], where the authors also formulate the problem in terms of time delay using a similar methodology. However, in that work the authors considered two simplifications: (*i*) the transcription and translation rate constants are uniform along the DNA and mRNA templates, respectively, and (*ii*) there may only be one RNA polymerase per DNA and one ribosome per mRNA. In contrast, we considered the case of non-uniform elongation rate constants and multiple ribosomes on the mRNA template.

Heinrich and Rapoport [23] proposed a delay model for the initiation rate, 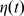, and the concentration of bound ribosomes, 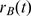, in the special case of constant initiation and termination rate constants, and constant elongation rate constants, equal for each codon. Our delay model is a generalization of theirs and reduces exactly to their model when subject to the same parameter restrictions (Text S4).

While the model by Heinrich and Rapoport offers the same advantages discussed above, our delay model is based on a systematic derivation from Eqs. 1 which ensures that all essential aspects are captured, and it permits the explicit estimation of the regimes of agreement with the full mechanistic model. In addition, our formulation offers an improvement over previous time-delay models. It accounts for average ribosome sequestration on mRNA chains and allows a good approximation of the ribosome distribution and dynamics. In our model the ribosome distribution may be obtained explicitly and so position dependent effects, such as codon usage and energetic considerations [80], may be studied.

### Computational Studies

We first performed a computational study in order to identify the ranges of the parameter values for which the time-delay model is in good agreement with the mechanistic model. We compared the dynamic responses to step changes and to periodic forcing of the initiation rate constant. These studies provide the necessary conditions for the successful application of time delay to the modeling and analysis of genetic networks which display complex dynamic behavior [12]–[16].

Throughout the computational studies in this section, we use parameters as in Table 3, chosen to be within the typical parameter ranges for *E. coli* (see Table 1).

**Table 3:** Parameters used for computational studies.

Notation	Description of dimensionless parameter	Value
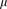	mRNA single species concentration	0.01^a^
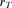	Total ribosome concentration	1^a^
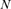	mRNA size	144 codons^b^
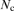	Characteristic mRNA size	144 codons^c^
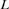	Ribosome length	12
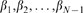	Elongation rate at codon 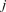	144^d^

*^a^*For a single mRNA species, the copy number in *E. coli* is on the order of 10. Due to competition with other messages, a single mRNA species is exposed only to an ‘effective’ ribosome concentration, 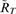, equal to the total free ribosomes, which is 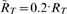 (see Table 1). Then 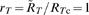 by choosing 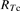 equal to this ‘effective’ ribosome concentration.

*^b^*Following [23], mRNA length used corresponds to the mean of 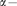 and 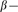 globin in reticulocytes.

*^c^*Value chosen to be equal to the mRNA size used.

*^d^*All dimensional elongation rate constants, 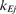, 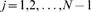, chosen to be equal to their characteristic value, 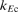. Thus, 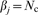 as the dimensionless elongation rates are defined as 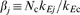.

#### Parameter domain for equivalence between models

The time-delay model was originally derived under the assumption of low ribosome density. Therefore, we first verified by simulation the validity of the our model for very low ribosome density and we identified the upper bound of ribosome density for which the responses of the delay model are in good agreement with those of the mechanistic model.

**Figure 2:**
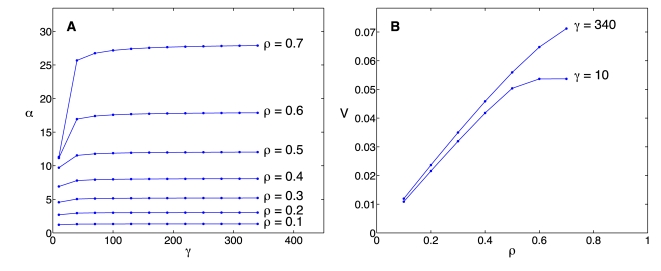
Ribosome density for given kinetic rate constants and ribosome flux as a function of density. (A) The loci of dimensionless termination (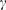) and initiation (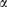) rate constants that yield a steady state solution with a given density, 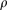. (B) Ribosome flux (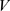) for steady state solutions as a function of ribosome density. For the two curves shown, 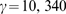, the initiation rate constant is approximately in the ranges 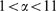 and 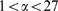, respectively. The remaining parameter values are 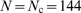, 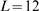, 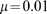, 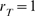 and 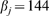 for 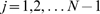.

We first mapped the values of the kinetic parameters of the mechanistic model into ribosome densities. We considered an mRNA species of fixed length 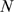 codons, and we assumed that the 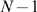 elongation rate constants were the same for each codon: 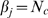. Under this assumption, the ribosomal density, 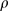, is a function of only two parameters: the initiation and termination rate constants, 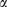 and 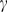 respectively. For each value of 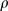 we calculated a unique curve in the 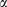-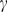 parameter space (Figure 2(A)), and along each curve, the ribosome flux, 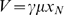, grows in the direction of increasing termination rate constant, 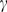. As a function of ribosomal density, the flux increases and reaches a maximum as ribosomal steric hindrance becomes limiting for protein production (Figure 2(B)).

The elongation rate constants are known to be different for each codon [28], [76]–[79] and this variation has interesting consequences in terms of ribosome organization [28]. However, for our present computational studies we make the simplifying assumption of uniform elongation rate constants. The agreement obtained between the mechanistic and time-delay models under this simplifying assumption will still hold in the case of variable elongation rates as long as the ribosomal distribution (*i*) has a low local density and (*ii*) is nearly uniform along the chain (Texts S2 and S3). These conditions will generally hold if initiation is limiting and if the elongation rates vary slowly enough along the chain and have big enough values to avoid ribosomal queuing.

The initiation and termination pairs with 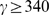 give maximum ribosome flux and those with 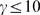 are said to give minimum flux. In terms of the ribosome distribution, parameters that yield maximum flux correspond to steady state distributions that are essentially uniform; as 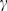 is lowered protein translation becomes increasingly termination limited and considerable ribosome packing results on the last codon.

#### Transient protein induction

**Figure 3:**
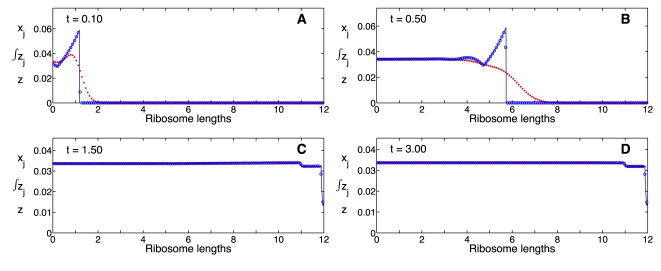
Ribosome distribution as a function of time. The ribosome distributions along the mRNA chain during induction, as predicted by the mechanistic model (Eqs. 10, dots), and the time delay model (Eqs. 21, open circles) at times (A) 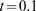, (B) 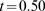, (C) 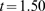 and (D) 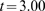. Parameter values are 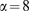, 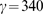, 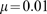, 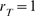, 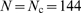, 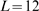 and 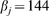 for 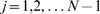. Distance along the chain is measured in ribosome lengths and the scale for the y-axis is different in each panel.

We studied the performance of the time-delay model in capturing the dynamics of protein induction, during which protein synthesis is initiated from a newly synthesized mRNA molecule which is not occupied initially by ribosomes. We first compared the number of ribosomes per codon at different time points predicted by the two models: the mechanistic model and the time-delay model (Figure 3). We used characteristic values of the initiation, elongation, and termination rate constants which yield a ribosome density 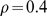. This value is close to the average ribosome density in *E. coli* [67]. The time-delay model captures very well the dynamics of the ribosome distribution along the mRNA, and it is in excellent agreement with the mechanistic model at steady state. It captures the density drop over the last ribosome length on the chain, resulting from the absence of interference between ribosomes over this last segment, see Figs. 3(C) and 3(D). However, the time-delay model develops a sharp front for the ribosome distribution, whereas the mechanistic model predicts a ribosome distribution that spreads out over time. This disagreement is expected because in the derivation of the time-delay model from the PDE model, we omitted the second and higher order diffusive terms (Text S2). The inclusion of these terms improves the agreement in the ribosome distribution between the two models (results not shown), however, including them impedes us from obtaining a practical time-delay model. Moreover, we find it unnecessary to introduce further corrections to our approximation, since the level of agreement of the two models is already very good in the physiological regime.

**Figure 4:**
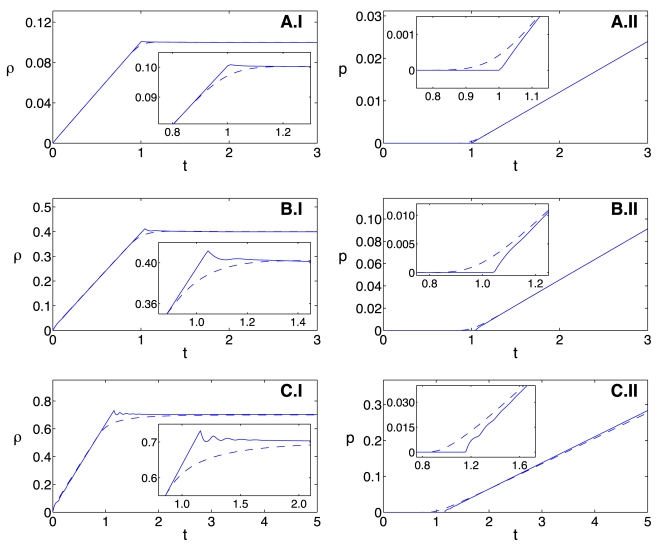
Ribosome density and protein concentration as functions of time. Numerical simulations, at high ribosome flux, of the ribosome density (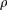) and protein concentration (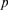) as functions of time; mechanistic model (Eqs. 10, dashed line), time-delay model (Eqs. 21, solid line). The initiation rates constants used are (A) 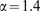, (B) 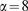 and (C) 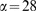, respectively. The remaining parameter values are 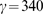, 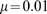, 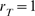, 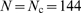, 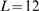 and 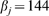 for 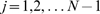.

**Figure 5:**
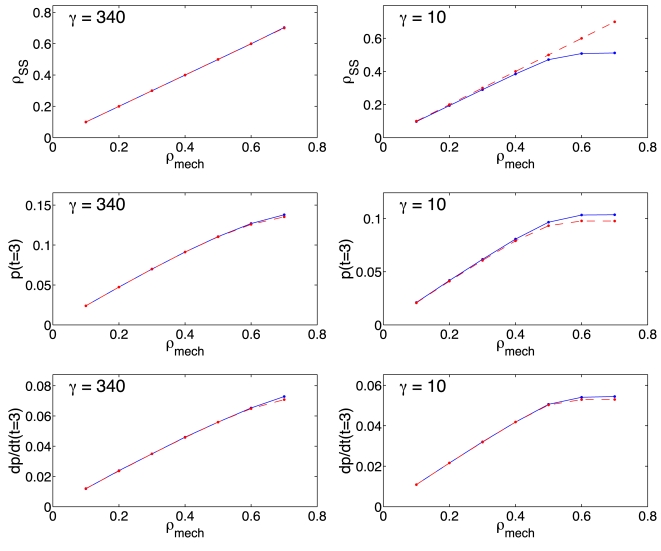
Steady state comparisons of the models. Steady state density (top), protein concentration (middle) and protein production rate (bottom) as functions of the steady state density of the mechanistic model, 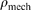. Mechanistic model (Eqs. 10) shown with the broken line, continuous line corresponds to results from delay model (Eqs. 21). Left panels: maximum flux (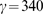) and 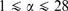. Right panels: minimum flux (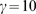) and 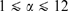. For the abscissas, the initiation rate constant grows in the direction of increasing density. Protein concentration (middle panels) and protein production rate (bottom panels) shown at the representative time of 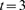. Other parameters: 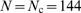, 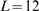, 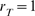, 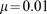 and 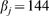 for 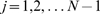.

While the distribution of ribosomes along an mRNA molecule is an important property of translation, the rate of protein synthesis, the protein levels, and the concentration of ribosomes are the quantities that couple different genes and hence represent the most important outputs of genetic networks. For this reason, we performed two comparisons of these quantities: *(i)* in a dynamic situation and *(ii)* at steady state. We compared the dynamics through simulations of the two models at high flux (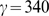), using different initiation rate constants, 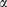, which correspond to steady state ribosome densities of 0.1, 0.4 and 0.7, and uniform ribosome distribution along the mRNA (Figure 4). The time-delay model presents discontinuities for both 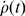 and 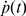 when the sharp front in the ribosome distribution reaches the end of the chain (Figure 3) and the first protein molecule is produced. This time is equal to the elongation time delay for the corresponding kinetic parameters. As expected, the mechanistic model predicts a smooth increase in the protein synthesis rate and protein levels due to the ribosome distribution spreading out; this leads to the production of proteins at times slightly shorter than the theoretical delay. The steady state density, protein levels, and protein synthesis rates predicted by the time-delay model are compared with those from the mechanistic model (Figure 5).

Based on the theory of the derivation of the time-delay model (Text S2), we expect good agreement between the time-delay and the mechanistic model, when the ribosomal density is low (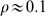), and the ribosomes are uniformly distributed along the mRNA (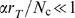 and 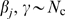). The computational studies presented suggest that excellent agreement between the two models is possible for ribosome densities as high as 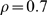, as long as the ribosomes are uniformly distributed (Figure 4 and left panels of Figure 5).

The nonuniform distribution of the ribosomes results in higher delay times, lower ribosome flux, and lower rates of protein synthesis. Under these conditions the accuracy of the time delay model decreases, even for ribosome densities 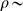
0.1, 0.2, as nonlinear terms neglected in the approximation become more significant (right panels of Figure 5). Under these conditions the interaction between a ribosome and the one preceding it become significant as ribosomes concentrate at the end of the chain due to slower termination rate constants, and the time required to reach steady state becomes vastly different between the models. Even at low ribosome densities, the mechanistic model requires one order of magnitude more time to reach the steady state, whereas the time-delay model reaches the steady approximately after a time equal to one elongation time delay (Eq. 19b, results not shown). Nevertheless, this is not a physiological condition since experimental results suggest that translation occurs with a nearly uniform ribosome distribution [19].

#### Protein production under time varying conditions

One of the objectives for the development of the time-delay model is to use it for the analysis of genetic networks that display complex dynamic behavior, such as oscillations [7]–[9]. In these networks, the expression of genes is regulated by the levels of regulatory proteins in the system. When the levels of the regulatory proteins oscillate, the protein synthesis experiences a time-dependent forcing. In addition, fast dynamics are expected in genetic circuits when stochastic factors are taken into account, and when bursts in expression occur [81],[82]. During these periods, the copy number of a protein may change rapidly in a time-scale of a few minutes and the network coupling may propagate perturbations on the same time-scale along the network. This leads us to study the time-delay model under time variable conditions. In our model reduction we allow the kinetic parameters to vary slowly in time around some mean value that is consistent with the conditions of nearly uniform and low density. We perform a simulation with an initiation rate constant that varies in time according to 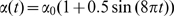, where the values chosen for 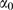 and 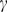 correspond to a steady state ribosome density of 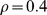 and the forcing period is about a quarter of the elongation time. Although the choice of frequency appears to be high relative to burst time-scales, it was chosen to test our approximations under extreme conditions, since it is expected that the discrepancies between the mechanistic and our time-delay model increase with increasing frequency.

**Figure 6:**
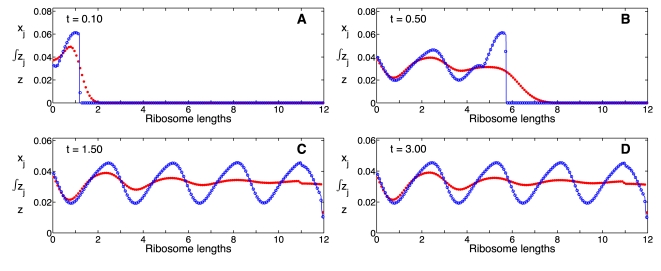
Ribosome distribution as a function of time with a time-varying initiation rate. Numerical simulation showing 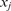 (dots) and 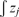 (open circles) starting from an empty mRNA chain at times (A) 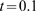, (B) 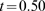, (C) 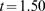 and (D) 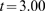. Parameters are 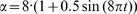, 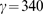, 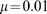, 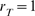, 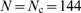, 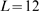 and 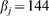 for 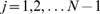. Note the change in the vertical scale in each panel. Distance along the chain is measured in ribosome lengths.

The forcing of the initiation rate causes a periodic loading of the mRNA which appears as a wave of the ribosome distribution. The evolution and dynamics of the ribosome distribution predicted by the time-delay and the mechanistic model are in good agreement near the initiation site (Figure 6(A)). In an experiment with uniform elongation rates, one expects some ribosomes to elongate at slightly different rates, due to stochastic effects, with the resulting ribosome distribution wave spreading out. This effect is captured by the mechanistic model, whereas the time-delay model predicts a wave with a constant period and amplitude along the template. The periods of oscillation for both the mechanistic and the time-delay models are, of course, approximately equal to the period of the initiation rate constant.

**Figure 7:**
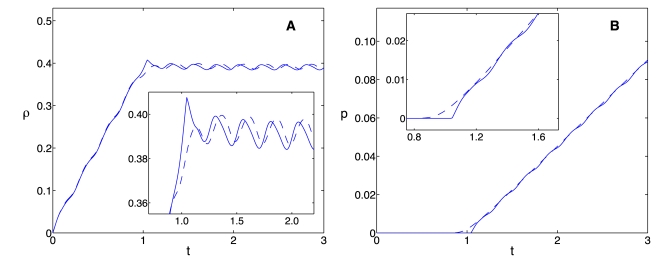
Protein concentration and ribosome density as functions of time with a time-varying initiation rate. (A) Ribosome density and (B) protein concentration as functions of time, mechanistic model shown with the dashed curve, approximate delay model shown with the continuous one. Parameters as in Figure 6.

Despite these differences the time-averaged performance of the two models is very similar (Figure 6(C,D)). This similarity is manifest in the excellent agreement of the dynamics of the ribosome density, the protein synthesis rate and the protein levels between the two models (Figure 7). The spreading of the ribosome distribution is again responsible for a small phase shift in the time-dependent ribosome density between the two models (Figure 7(A)), and the earlier onset of protein synthesis in the mechanistic model (Figure 7(B)). These discrepancies appear during the earlier times because the forcing starts with an empty mRNA molecule, but they are reduced significantly at longer time scales.

#### Oscillatory behavior in a self-repressing gene

We next test the ability of our time-delay model to capture behavior around a bifurcation point, where the qualitative behavior of the system changes dramatically as a parameter is varied. We choose for this test a gene with negative feedback transcription regulation, i.e. a self-repressing gene. As the protein expression time delay increases, this system is able to transition from a stable fixed point to self-sustained oscillations [7]–[9]. We here show that purely translational time-delays can also drive this type of behavior and that it is well captured by our time-delay model.

We combine our proposed framework (Texts S2 and S3) with a commonly used heuristic model [7]–[9] to describe the self-repressing gene by the following time-delay model:
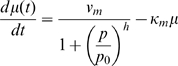
(22a)

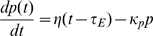
(22b)

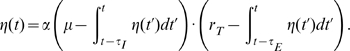
(22c)The negative feedback in the circuit is modeled by a Hill function with parameters 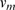, 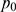 and 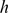 that are greater than zero. The mRNA and protein degradation rates are 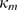 and 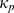, respectively. Although it can be shown that this model overestimates the protein synthesis rate with respect to models that consider mRNA degradation more carefully, we nevertheless adopt it as an approximation and focus on a regime where this will not alter our conclusions. This model may be transformed into a delay differential equation as was done in Eq. 21.

Our time-delay model is particularly well suited to study the system's temporal asymptotic behavior as a function of the translation time delay, 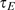, since this quantity appears as an explicit parameter in the model. We use a numerical bifurcation package [66] to study the effect of increasing the translational time delay by increasing the codon number of the mRNA templates. For the smaller codon numbers considered, the model has a stable fixed point, while at a codon number of about 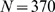, a Hopf bifurcation occurs and the system undergoes sustained oscillation for larger codon numbers.

We now test the agreement of our time-delay model for the self-repressing gene with the following mechanistic model for the same system:
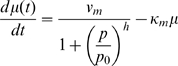
(23)with the protein synthesis dynamics described by the following equations
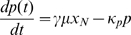
(24a)

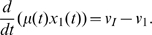
(24b)

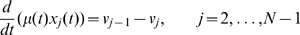
(24c)

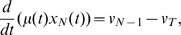
(24d)with 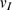, 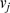, for 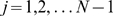 and 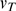 given in Eq. 11.

**Figure 8:**
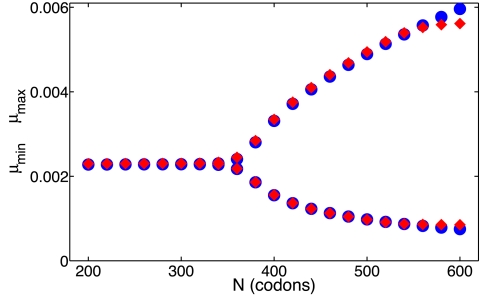
Maxima and minima of the mRNA concentration for different codon numbers. The maxima and minima of the mRNA concentration are shown after transients have decayed. The result from the mechanistic model is shown with crosses, the time-delay model is shown with circles. The two models undergo a Hopf bifurcation near a codon number of 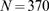, at this point the behavior changes from steady-state decay to oscillatory. Parameters are 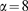, 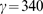, 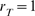, 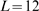, 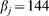 for 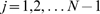, 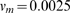, 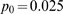, 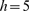, 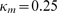 and 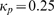.

It is not possible to use the codon number as a bifurcation parameter of the model in Eqs. 23 and 24, since 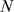 is not an explicit parameter. However, we simulate both models for values of the chain size between 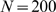 and 600 and we monitor the maxima and minima of the mRNA concentration after the decay of transients. The results show the Hopf bifurcation at a codon number of about 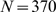 that was seen (Figure 8). The time-delay model captures with great precision the codon number at which the Hopf bifurcation occurs. Furthermore, our model also agrees with the full mechanistic model on the precise values of the mRNA concentration extrema. The oscillations of this system are driven by the translational time-delay and have a period between 5–10 times the time delay (results not shown).

This shows the ability of our time-delay model to capture the behavior of the mechanistic model during a bifurcation. Our formulation provides conceptual advantages by stressing the importance of the elongation time-delay as the driving force behind the oscillations. In contrast with the mechanistic model, in our time-delay model the mRNA codon number is a parameter that may be used for bifurcation studies. The time-delay model allows performing novel studies without the need of additional parameter fitting.

This analysis allows us to identify two future areas of study. First, the mechanistic model of Heinrich and Rapoport must be extended to include a detailed description of mRNA degradation, from which we could formulate a time-delay model, derived from the mechanistic model. Then, the extended model must be analyzed in detail, using methods from nonlinear dynamics such as bifurcation analysis, to identify the regions of parameter space with different behaviors. Our preliminary analysis has shown that it is possible for a single gene to display oscillatory dynamics driven by time-delays, without the need of additional regulatory components [16]. For oscillations to be feasible, we require mRNA and protein half-lives comparable to each other and to the time-delay, as has been noted by other authors [9],[16]. The extended studies will provide more thorough design criteria for gene regulatory networks to display complex dynamic behavior such as bi-stability and limit cycles.

## Discussion

We developed a rigorous methodology that allows the formulation of a reduced, time-delay model of protein synthesis based on a detailed mechanistic model. The systematic reduction of the mechanistic model allows the exact mapping between its parameters into the parameters of the time-delay model, and it provides analytical evaluation of the differences between the two models.

Theoretical and computational analysis suggests that the time-delay model is an excellent approximation of the mechanistic model under conditions that correspond to *(i)* a uniform distribution of the ribosomes along the mRNA molecule and *(ii)* a ribosome density below 0.5. It has been shown that these conditions can be achieved under initiation limiting conditions [27],[28], and they are indeed the physiological conditions in *E. coli* and yeast.

In our continuing studies we have used the time-delay model to perform bifurcation analysis of genetic networks having feedback mechanisms operating at the transcriptional level. Such analysis would not be practical using the full mechanistic model and tests the limits of performance of the reduced time-delay model under complex dynamic behavior. In genetic networks, interactions and feedback mechanisms operate through protein concentrations. In turn, the mRNAs in the circuit compete for free ribosomes, essential to produce protein for the interaction circuits. Hence, the important quantities to consider are precisely the protein concentration as well as the amount of free and bound ribosomes. In this context, the good agreement obtained for the ribosome density and protein concentration, 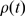 and 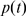, is quite significant.

In the study of protein translation, we have identified three main areas of future developments: *(i)* the modeling of the variable elongation rate constant, *(ii)* the modeling of sequence specific degradation of mRNA and *(iii)* the modeling of mRNA secondary structure effects. It has been shown that these three elements are important for the steady-state and dynamic properties of genetic networks, therefore, a rigorous description of these processes in time-delay models is challenging but very important.

Mathematical models of stochastic systems may be reduced to models with time delays by lumping some of the intermediate processes [53]–[56]. However, this reduction is usually done in a heuristic way by assuming that the products of some reactions appear in the mixture after a certain discrete time delay. In our formulation the time-delays emerge through systematic approximations on a mechanistic model with no time delays. We could build on previous work by Roussel and Zhu [54], where they obtained the time delay distribution by explicitly modeling the equivalent steps and quantifying the time they require. If the time delay distribution is sharply peaked, then we could lump the series of processes and substitute them by a fixed time delay. To properly apply this procedure one should ensure that the system stays within the approximation's regime of validity. However, determining when one should use deterministic delay equations or non-Markov stochastic models under general settings is a difficult question that requires careful investigation. This represents an area of interesting future research.

The method used here may be useful for developing reduced, time-delay models of mRNA transcription, since the underlying biophysical and biochemical phenomena are very similar. Both processes involve molecular machines scanning a template in order to build a polymer chain [83]–[85]. A great number of modeling studies have contributed to our understanding of the process of transcription. These investigations can be grouped in two general classes. First, there are investigations that use an approach based on chemical kinetics and thermodynamics to obtain information about the process at a molecular level [86]–[88]. Alternatively, some studies use mathematical models that lump certain molecular details and parameters together and are useful to understanding the problem at a larger scale. These lumped models of transcription are useful for describing various processes, such as: mRNA degradation, the dynamics of simple genetic circuits, the variability of transcription elongation times, the accuracy of reduced vs. more detailed models, etc. [20],[54],[89],[90]. However, many lumped models have been constructed based on ad hoc assumptions without a systematic model reduction.

Some differences do exist between the processes of transcription and translation. One has to do with the existence of stall stages at certain sequence positions, where RNA-polymerases may stop for as long as several seconds and generate queueing of several polymerases behind the stalled one [85],[91]. However, except at stall sites, steric hindrance is weaker than in translation (a density of RNA-polymerases of 0.25 was measured experimentally in the *lacZ* message of *E. coli*, [69]), though the possibility of multiple polymerases transcribing the same gene should still be considered. A mechanistic model similar to the one of Heinrich and Rapoport [23] may be developed to study transcription, and it could capture polymerase stalling by having small elongation rates at the stall sites. In situations where stalling is not severe, a reduced, time-delay model may be applied with confidence to this problem, following our methodology.

In summary, given the complexity and the importance of transcription and translation in biological processes, it is necessary to develop methodologies to systematically reduce detailed mechanistic models of these processes. To reach this objective, the formulation of time-delayed models of coupled template polymerization processes is one of the exciting future developments in the modeling of genetic networks.

## Supporting Information

Text S1 Estimation of the Non-Dimensional Initiation Rate Constant
(0.03 MB PDF)

Text S2 Derivation of the PDE Model
(0.07 MB PDF)

Text S3 Integral equation formulation
(0.07 MB PDF)

Text S4 Equivalence with a Previously Proposed Delay Model
(0.03 MB PDF)

Text S5 The Steady State Solution and its Stability
(0.04 MB PDF)
