# Supplementary material for: The Origins of Time-Delay in Template Biopolymerization Processes
Source: PLoS Comput Biol. 2010 Apr 1;6(4):e1000726. doi: 10.1371/journal.pcbi.1000726 (PMC2848540; doi:10.1371/journal.pcbi.1000726)
Supplement: Text S1 — Estimation of the Non-Dimensional Initiation Rate Constant (0.03 MB PDF) [file pcbi.1000726.s001.pdf]

Text S1 Estimation of the Non-Dimensional Initiation Rate Constant

An estimate for the value of the non-dimensional initiation rate constant is readily obtained from biological considerations. Under the typically predominant initiation limited conditions [18,19], at steady state, the ribosomes are uniformly distributed along the mRNA chain with low interference. Ribosomal densities measured experimentally give $\rho \sim 0.3$ in *E. coli* and $\rho \sim 0.2$ in *S. cerevisiae* [18,67]. For the derivation of our time-delay model, we use an estimate of the initiation rate constant assuming a situation of ribosome excess and use parameter values as in Table 3, chosen to be within the physiological range of parameters (Table 1).

First, we assume a nearly uniform distribution, $x_j \sim \bar{x}$, $\rho \sim L\bar{x}$, a typical density $\rho \sim 0.3$ in *E. coli*, and note that the concentration of bound ribosomes is negligible with respect to the total concentration. Explicitly, $r_B = \mu \sum_{s=1}^{N} x_s = \mu \frac{N}{L} \rho \sim 10^{-2} \frac{144}{12} 0.3 = 0.04$, which is much smaller than $r_T = 1$. Thus, in the case of great ribosome excess: $r_F = r_T - r_B \simeq r_T$.

Using the above information, we estimate the time averaged initiation rate constant. From the dimensionless mechanistic model, Eqs. 10, we have for the steady state of the first codon

$$\langle \alpha \rangle \left(1 - \sum_{s=1}^{L} x_s \right) \langle r_F \rangle = \langle \beta_1 \rangle x_1 \frac{1 - \sum_{s=1}^{L} x_{1+s}}{1 - \sum_{s=1}^{L-1} x_{1+s}}, \tag{S1.1}$$

where $\langle \cdot \rangle$ denotes time average. We again assume a nearly uniform distribution, $x_j \sim \bar{x}$, $\rho \sim L\bar{x}$, a density $\rho \sim 0.3$ and $\langle \beta_j \rangle \sim N_\mathrm{c}$ from Eq. 14.

Then, independently of the ribosome abundance

$$\frac{\langle \alpha \rangle \langle r_F \rangle}{N_\mathrm{c}} \sim \frac{\rho / L}{1 - \frac{L-1}{L} \rho} = 0.03. \tag{S1.2}$$

In the case of great ribosome excess, $r_F = r_T - r_B \simeq r_T$, we get from Eq. S1.2

$$\frac{\langle \alpha \rangle \langle r_T \rangle}{N_\mathrm{c}} \sim \frac{\rho / L}{1 - \frac{L-1}{L} \rho} = 0.03. \tag{S1.3}$$

This is the small parameter used in our perturbation expansion in Text S2.

As mentioned, our time-delay model is derived in the case of great ribosome excess. However, it is possible to derive the model in the case of non-ribosome excess in a similar way. In this case, one uses $r_F = 0.2 \cdot r_T$ in Eq. S1.2 instead of $r_F \ll r_T$ (Table 1). The parameter combination $\langle \alpha \rangle \langle r_T \rangle / N_\mathrm{c}$ will be of a different magnitude but it equally serves as a small parameter for an equivalent perturbation expansion.
