# Supplementary material for: The Origins of Time-Delay in Template Biopolymerization Processes
Source: PLoS Comput Biol. 2010 Apr 1;6(4):e1000726. doi: 10.1371/journal.pcbi.1000726 (PMC2848540; doi:10.1371/journal.pcbi.1000726)
Supplement: Text S2 — Derivation of the PDE Model (0.07 MB PDF) [file pcbi.1000726.s002.pdf]

Text S2 Derivation of the PDE Model

In this section we derive an approximation to the mechanistic model of Heinrich and Rapoport [23], for a ribosome distribution with low and nearly uniform amplitude, conditions satisfied physiologically [18]. In this case, the number of ribosomes over a ribosome length at any position on the chain is small compared to one, namely $\sum_{j=1}^{L} x_{s+j} \ll 1$ and thus $x_j \ll 1/L$, for all j's. Numerical work [23], has shown that if initiation is low enough, there exist steady state solutions satisfying these requirements. In addition, for the derivation of our time-delay model we assume a situation of ribosome excess as discussed in Text S1.

Using the non-dimensional variables from the Results section, ribosome dynamics on mRNA chains are modeled by

$$\mu\frac{d}{dt}(x_1) = v_I - v_1, \tag{4a}$$

$$\mu\frac{d}{dt}(x_j) = v_{j-1} - v_j, \qquad j = 2, \ldots, N \tag{4b}$$

where the non-dimensional fluxes are

$$v_I = \alpha\mu\left(1 - \sum_{s=1}^{L} x_s\right)\left(r_T - \mu\sum_{s=1}^{N} x_s\right), \tag{5a}$$

$$v_j = \mu\beta_j x_j \frac{1 - \sum_{s=1}^{L} x_{j+s}}{1 - \sum_{s=1}^{L-1} x_{j+s}}, \quad j = 2, \ldots, N - L, \tag{5b}$$

$$v_j = \mu\beta_j x_j, \qquad j = N - L + 1, \ldots, N - 1, \tag{5c}$$

$$v_T = \mu\gamma x_N, \qquad j = N. \tag{5d}$$

The parameters r_T, α, β_j's and γ are allowed to be time-dependent. Additionally, for notational convenience, from here on we will denote γ by β_N.

We use a perturbative expansion to obtain a simplified version of the model given by Eq. S2.1, in which the solution is given by a slowly-varying quasi-steady state plus deviations of smaller order. We obtain thus a systematically derived approximation which keeps track of the order of the error.

To carry out the expansion we introduce a small parameter ϵ. In the situation of great ribosome excess (Text S1), we may disregard the depletion of free ribosomes and $\alpha r_T/N_c$ is an estimate of the number of ribosomes per mRNA initiating in a time $1/N_c$. This is the time required to advance one codon, and so $x_1 \sim \alpha r_T/N_c$. A small parameter giving the magnitude of initiation is thus defined as

$$\epsilon^2 \equiv \frac{\langle\alpha\rangle\langle r_T\rangle}{N_c}, \tag{6}$$

where $\langle\cdot\rangle$ represents a time average. It is shown here that under suitable conditions, this gives rise to solutions of amplitude $x_j \sim \epsilon^2$.

We choose the scaling of L, $L \sim \epsilon^{-1}$, to satisfy low ribosome packing, $\sum_{r=1}^{L} x_{j+r} \ll 1$, or $L\epsilon^2 \ll 1$. The mRNA size is taken as $N, N_c \sim \epsilon^{-2}$, and so $\beta_j \sim \epsilon^{-2}$ since $\beta_j = N_c \cdot \mathcal{O}(1)$. Finally from biological evidence, $1/L$, $\mu \sim 1/10$, so for our investigations the dimensionless mRNA concentration, μ, is assumed to be $\mu \sim \epsilon$. In order to work with $\mathcal{O}(1)$ quantities, the following scalings are used

$$\beta_j = \epsilon^{-2}\hat{\beta}_j, \qquad \mu = \epsilon\hat{\mu}, \tag{7}$$

where quantities with a circumflex are $\mathcal{O}(1)$.

Two timescales are introduced

$$t_0 = t, \qquad t_{-2} = \epsilon^{-2}t, \tag{8}$$

the first represents the timescale on which the whole elongation process takes place, whereas the second one is the fast time of order $1/N$ on which the elongation of one single codon happens. If the parameters of the problem vary only on the long timescale, i.e., $\frac{\dot{\alpha}}{\alpha}$, $\frac{\dot{\beta}_j}{\beta_j}$, $\frac{\dot{r}_T}{r_T} \sim 1$, then slowly varying, quasi-steady state solutions $x_{qss\ j}$ exist, obtained by solving

$$v_I = v_1, \qquad v_j = v_{j-1}, \quad j = 2, 3, \ldots N \tag{9}$$

for the $x_{qss\ j}$'s in terms of the slowly time varying parameters. Approximate solutions may be found to these nonlinear algebraic equations by using a perturbation expansion

$$x_{qss\ j} = \epsilon^2 x^{(2)}_{qss\ j} + \epsilon^3 x^{(3)}_{qss\ j} + \ldots \tag{10}$$

in the equations, such that the first few terms are

$$x^{(2)}_{qss\ j} = \frac{\hat{\beta}_1}{\hat{\beta}_j} x^{(2)}_{qss\ 1}, \qquad x^{(3)}_{qss\ j} = \frac{\hat{\beta}_1}{\hat{\beta}_j} x^{(3)}_{qss\ 1}, \tag{11}$$

with $x^{(2)}_{qss\ 1}$ and $x^{(3)}_{qss\ 1}$ expressed as

$$x^{(2)}_{qss\ 1} = \frac{\alpha r_T}{\hat{\beta}_1}, \tag{12a}$$

$$x^{(3)}_{qss\ 1} = -\frac{\alpha}{\hat{\beta}_1} x^{(2)}_{qss\ 1} \left[r_T \epsilon \sum_{r=1}^{L} \frac{\hat{\beta}_1}{\hat{\beta}_r} + \epsilon^2 \hat{\mu} \sum_{r=1}^{N} \frac{\hat{\beta}_1}{\hat{\beta}_r} \right]. \tag{12b}$$

Note that since $L \sim \epsilon^{-1}$ and $N \sim \epsilon^{-2}$, sums of $\mathcal{O}(1)$ quantities of the form $\epsilon \sum_{r=1}^{L}$ and $\epsilon^2 \sum_{r=1}^{N}$ are $\mathcal{O}(1)$.

We study solutions that are close to the slowly-varying quasi-steady state with deviations from it that depend on the fast timescale

$$x_j(t) = x_{qss\ j}(t_0) + \tilde{x}_j(t_{-2}), \tag{13}$$

where, as shown above, $x_{qss\ j} \sim \epsilon^2$ and the deviation is assumed to be small, $\tilde{x}_j \sim \epsilon^3$. When expressing the time derivative of this function as $\dot{x}_j(t) = \partial_{t_0} x_{qss\ j}(t_0) + \epsilon^{-2} \partial_{t_{-2}} \tilde{x}_j(t_{-2})$ and inserting the expansions

$$x_{qss\ j} = \epsilon^2 x^{(2)}_{qss\ j} + \epsilon^3 x^{(3)}_{qss\ j}, \tag{14a}$$

$$\tilde{x}_j = \epsilon^3 \tilde{x}^{(3)}_j + \epsilon^4 \tilde{x}^{(4)}_j, \tag{14b}$$

into Eqs. S2.1, several terms cancel out due to the construction of the functions $x_{qss\ j}$. After dividing by μ on both sides, the terms of lowest order that remain are $\mathcal{O}(\epsilon)$ and satisfy the linear ODE problem:

$$\partial_{t_{-2}} \tilde{x}^{(3)}_1 = -\hat{\beta}_j \tilde{x}^{(3)}_1, \tag{15a}$$

$$\partial_{t_{-2}} \tilde{x}^{(3)}_j = \hat{\beta}_{j-1} \tilde{x}^{(3)}_{j-1} - \hat{\beta}_j \tilde{x}^{(3)}_j, \qquad j = 2, 3, \ldots N. \tag{15b}$$

The associated matrix of this linear problem has purely negative eigenvalues and so solutions decay exponentially. To $\mathcal{O}(\epsilon^2)$:

$$\partial_{t_{-2}} \tilde{x}^{(4)}_1 = -\hat{\beta}_1 \tilde{x}^{(4)}_1 - \partial_{t_0} x^{(2)}_{qss\ 1} - \alpha \hat{\mu} \epsilon^2 \sum_{r=1}^{N} \tilde{x}^{(3)}_r - \alpha r_T \epsilon \sum_{r=1}^{L} \tilde{x}^{(3)}_r, \tag{16a}$$

$$\partial_{t_{-2}} \tilde{x}^{(4)}_j = \hat{\beta}_{j-1} \tilde{x}^{(4)}_{j-1} - \hat{\beta}_j \tilde{x}^{(4)}_j - \partial_{t_0} x^{(2)}_{qss\ j}, \quad j = 2, 3, \ldots N. \tag{16b}$$

The homogeneous component has the same matrix as before and so has purely negative eigenvalues while the forcing terms are $\mathcal{O}(1)$. Thus, there is no exponential blow-up of solutions.

An analysis of the linear system in Eq. S2.12 shows that although all the $\tilde{x}_j^{(3)}$ decay to zero on the fast scale t_{-2}, the effect of a nonzero initial condition in $\tilde{x}_1^{(3)}$ reaches the end of the chain only after a time $t_0 \sim 1$. We use the approximation $\tilde{x}_1^{(3)} = 0$ which produces an error of order $\mathcal{O}(\epsilon^3)$ in x_1 while still taking into account the time delay due to information propagating down the chain.

In summary, the approximation to be used in the dynamical equations is

$$\mu \frac{d}{dt}(x_j) = v_{\text{app } j-1} - v_{\text{app } j} + \mathcal{O}(\epsilon^4), \quad j = 2, \ldots, N \tag{17}$$

where the approximate fluxes $v_{\text{app } j}$ are simply expressed by defining modified rate constants, β_j^*:

$$v_{\text{app } j} = \mu \beta_j^* x_j \qquad j = 1, 2 \ldots N \tag{18}$$

where

$$\beta_j^* = \beta_j \frac{1 - \sum_{r=1}^{L} x_{qss\ j+r}}{1 - \sum_{r=1}^{L-1} x_{qss\ j+r}}. \tag{19}$$

The dynamics of x_1 in the fast scale are neglected, and this function is then determined from the quasi-steady state ‘boundary condition’:

$$\mu \beta_1^* x_1 = v_I + \mathcal{O}(\epsilon^2) \tag{20}$$

with

$$v_I = \alpha\mu \left(1 - \sum_{r=1}^{L} x_r\right)\left(r_T - \mu \sum_{r=1}^{N} x_r\right). \tag{21}$$

From the order of the terms dropped, one concludes that the dynamics of the x_j’s are resolved to the first term only, $\mathcal{O}(\epsilon^2)$; in contrast, steady states, when parameters are in fact time-independent, are resolved to three terms, that is, up to $\mathcal{O}(\epsilon^4)$.

Our next step is to replace the functions $x_j(t)$, equal to the number of ribosomes per codon, by a continuous distribution via the transformation

$$x_j(t) = \int_{j-1}^{j} z(s,t)ds. \tag{22}$$

Here $z(s,t)$ is equal to the number of ribosomes per unit length of the mRNA. The discrete index which labels the codons, $j = 1, 2, \ldots N$, is replaced by a continuous variable s, $0 \le s \le N$; codon j corresponds to the segment $j - 1 < s < j$ of the complete domain.

The modified elongation rate constant, $\beta_j^*(t)$, is also extended to a continuous version, $c_E(s,t)$, such that $c_E(j,t) \approx \beta_j^*(t)$. This new function is referred to as a velocity function, being related to how fast ribosomes advance on the chain.

The rate constants β_j are chosen to be slowly varying from codon to codon in such a way that the velocity function has the properties:

$$c_E \sim \epsilon^{-2}, \quad \partial_s c_E \sim \epsilon^{-1}, \quad \partial_{ss} c_E \sim 1. \tag{23}$$

Slowly varying rate constants yield a quasi-steady state which is also slowly varying along the chain, see Eq. S2.8. We further assume that the ribosome distribution is sufficiently close to the slowly-varying quasi-steady state such that

$$z \sim \epsilon^2, \quad \partial_s z \sim \epsilon^3, \quad \partial_{ss} z \sim \epsilon^4. \tag{24}$$

Then

$$x_j = \int_{j-1}^{j} z ds = z(j,t) - \frac{1}{2}\partial_s z(j,t) + \mathcal{O}(\epsilon^4). \tag{25}$$

The mean value theorem is used to define points s_j^*, $s_j^{**} \in (j-1, j)$ such that

$$\begin{aligned} &\beta_{j-1}^* x_{j-1} - \beta_j^* x_j \\ &\quad = -\partial_s(c_E z)|_{s_j^*} + \frac{1}{2}\partial_s(c_E \partial_s z)|_{s_j^{**}} + \mathcal{O}(\epsilon^2) \\ &\quad = -\partial_s(c_E z)|_{s_j^*} + \mathcal{O}(\epsilon^2). \end{aligned} \tag{26}$$

Moreover, since $x_j = z|_{s_j^*} + \mathcal{O}(\epsilon^3)$, one has, after using Eq. S2.23 in the dynamical Eqs. S2.14

$$\partial_t z|_{s_j^*} = -\partial_s(c_E z)|_{s_j^*} + \mathcal{O}(\epsilon^2). \tag{27}$$

For the boundary condition of Eq. S2.17, $x_1 = z|_{s=0} + \mathcal{O}(\epsilon^3)$ and $\beta_1^* = c_E|_{s=0} + \mathcal{O}(\epsilon^{-1})$ are used:

$$\begin{aligned} c_E(0,t)z(0,t) =& \alpha\left(1 - \int_0^L z ds\right)\left(r_T - \mu\int_0^N z ds\right) \\ &+ \mathcal{O}(\epsilon) \end{aligned} \tag{28}$$

To assess the quality of the approximations, one takes z as $z = z_{qss}(s,t_0) + \tilde{z}(s,t_{-2})$, where the quasi-steady state is $\mathcal{O}(\epsilon^2)$ and the deviation from it is order $\mathcal{O}(\epsilon^3)$. Using this in Eqs. S2.24 and S2.25 reveals that both dynamics and steady state solutions of $z(s,t)$ are only resolved up to $\mathcal{O}(\epsilon^2)$. The accuracy of steady states would be improved if the velocity function had derivatives that vanished to a higher order. Additionally, in the case of time-independent parameters, the quasi-steady state is a true steady state and from its linear stability (see next section), small perturbations from it decay to zero.

It is necessary to verify that Eq. S2.21 is consistent with the dynamics given by the approximate model obtained. Analyzing the evolution of z, $\partial_s z$ and $\partial_{ss} z$ along the characteristics of Eq. S2.24 reveals that these three quantities evolve exponentially with a growth rate given by $-\partial_s c_E$ and so exponential growth results if $\partial_s c_E < 0$. In order to avoid a growth of this order, in addition to $\partial_s c_E \sim \epsilon^{-1}$ it is required that this function averages out to a non-negative value throughout the length of the chain. From a physical point of view of ribosomes moving along mRNA chains, this has the simple interpretation that ribosomes tend to pile up at places where the velocity decreases and sufficient pile-up would render the low density approximation invalid.

Extending Eq. S2.24 to the whole domain and putting everything together, the approximate PDE model takes the form

$$\partial_t z(s,t) + \partial_s\left(c_E(s,t) z(s,t)\right) = 0, \tag{29a}$$

$$\text{for} \quad 0 < s < N, \quad 0 < t$$

$$z(s,0) = z_0(s), \qquad 0 \le s \le N, \tag{29b}$$

$$z(0,t) = z_1(t), \qquad t > 0, \tag{29c}$$

with

$$\begin{aligned} c_E(0,t)z_1(t) = \alpha(t)&\left(r_T(t) - \mu\int_0^N z(s,t) ds\right) \\ &\cdot\left(1 - \int_0^L z(s,t) ds\right). \end{aligned} \tag{30}$$

The function $z_0(s)$ is the initial distribution of ribosomes on the mRNAs.

The ribosomal density at the $s = 0$ boundary is not known a priori, but must be determined by the boundary condition of Eq. S2.27. The initiation rate, $\eta(t) \equiv \alpha(t)\mu \left(r_T(t) - \mu \int_0^N zds \right) \left(1 - \int_0^L zds \right)$, is proportional both to the concentration of free ribosomes, $r_T(t) - \mu \int_0^N zds$ and to the concentration of mRNA chains with free initiation sites, $\left(1 - \int_0^L zds \right) \cdot \mu$, since it deals with a bimolecular reaction. The constant of proportionality is the initiation rate constant, $\alpha(t)$.

We emphasize at this point that if the parameters of the problem are not chosen in a way such that the original model gives low, slowly varying ribosomal density, then the continuum approximation may give unphysical results. For certain velocity functions $c_E(s, t)$ and α big enough, it is possible to obtain a distribution with more ribosomes on the chain than the ones that actually fit, N/L, as ribosome interaction is not taken into account in our PDE model.

However, the form of the boundary condition guarantees that regardless of parameter values and velocity function the following hold: (i) the density $z(s, t)$ is always positive, (ii) the number of ribosomes on the initiation site per mRNA is always less than one, $\int_0^L zds < 1$ and finally, (iii) the number of ribosomes bound to all chains is less than the total number of them available $\mu \int_0^N zds < r_T$.
