# Supplementary material for: The Origins of Time-Delay in Template Biopolymerization Processes
Source: PLoS Comput Biol. 2010 Apr 1;6(4):e1000726. doi: 10.1371/journal.pcbi.1000726 (PMC2848540; doi:10.1371/journal.pcbi.1000726)
Supplement: Text S3 — Integral equation formulation (0.07 MB PDF) [file pcbi.1000726.s003.pdf]

# Text S3 Integral equation formulation

In this section, we transform the PDE problem, Eqs. 16, obtained in Text S2 into an integral equation for the initiation rate, $\eta(t)$, by using the method of characteristics [?].

### Solution by the Method of Characteristics

For the problem in Eqs. 16, characteristic curves (those that satisfy $ds/dt = c_E(s,t)$) divide the $(s,t)$ plane into two regions: characteristic curves that meet the $t = 0$ boundary have initial values determined by $z(s,0) = z_0(s)$ while those that meet the $s = 0$ boundary have values determined from $z(0,t) = z_1(t)$. The curve $s(t)$ between the two regions satisfies $ds/dt = c_E(s,t)$ with $s(0) = 0$, see Fig. S1.

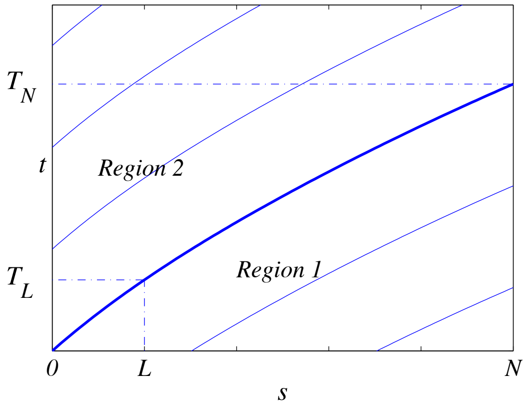


**Figure 1.** Characteristic curves on the $(s,t)$ plane, displaying the two times $T_L$ and $T_N$.

### Region 1

The system of equations to be solved is

$$\frac{dz}{d\zeta_1} = -z\partial_s c_E(s,t), \qquad z|_{\zeta_1=0} = z_0(\xi_1), \tag{28a}$$

$$\frac{ds}{d\zeta_1} = c_E(s,t), \qquad s|_{\zeta_1=0} = \xi_1, \tag{28b}$$

$$\frac{dt}{d\zeta_1} = 1, \qquad t|_{\zeta_1=0} = 0, \tag{28c}$$

$\zeta_1$ parameterizes displacement along the characteristics and $\xi_1$ labels the different characteristic curves.

Let $s = S_1(\xi_1,\zeta_1)$ and $t(\xi_1,\zeta_1) = \zeta_1$ be the solutions to Eqs. S3.1b and S3.1c. These two relations are to be inverted, in theory at least, to obtain $\xi_1 = \xi_1(s,t)$ and $\zeta_1 = \zeta_1(s,t)$. The solution is then:

$$z(s,t) = z_0(\xi_1) \cdot \exp\left\{-\int_0^{\zeta_1} \partial_s c_E(S_1(\xi_1,\zeta_1'),\zeta_1')d\zeta_1'\right\}, \tag{29}$$

where it is understood that $\xi_1$ and $\zeta_1$ are written as functions of $s$ and $t$.

**Region 2**

The characteristic equations in this region are

$$\frac{dz}{d\zeta_2} = -z\partial_s c_E(s,t), \qquad z|_{\zeta_2=0} = z_1(\xi_2), \tag{30a}$$

$$\frac{ds}{d\zeta_2} = c_E(s,t), \qquad s|_{\zeta_2=0} = 0 \tag{30b}$$

$$\frac{dt}{d\zeta_2} = 1, \qquad t|_{\zeta_2=0} = \xi_2, \tag{30c}$$

as before, $\zeta_2$ parameterizes displacement along the characteristics and $\xi_2$ labels each one of them.

We have $s = S_2(\xi_2, \zeta_2)$ and $t(\xi_2, \zeta_2) = \zeta_2 + \xi_2$ as solutions to Eqs. S3.3b and S3.3c, these are to be inverted to obtain $\xi_2 = \xi_2(s,t)$ and $\zeta_2 = \zeta_2(s,t)$ in region 2. The solution for $z(s,t)$ is then:

$$z(s,t) = z_1(\xi_2) \cdot \exp\left\{ -\int_0^{\zeta_2} \partial_s c_E(S_2(\xi_2, \zeta_2'), \zeta_2' + \xi_2) d\zeta_2' \right\}, \tag{31}$$

where $\xi_2$ and $\zeta_2$ are to be written as functions of $s$ and $t$.

The exponential factors that appear in the solutions of Eqs. S3.2 and S3.4, reflect the piling and unpiling of ribosomes due to spatial gradients in the velocity. This effect is absent for a velocity of the form $c_E(t)$, $\partial_s c_E = 0$, since all ribosomes on the mRNA speed up or slow down at the same rate.

**Transformation into an Integral Equation for $z_1(t)$.**

The solution in region 2 requires that $z_1(t)$ be chosen to satisfy the boundary condition of Eqs. 16 at $s = 0$. Carrying out this process yields an integral equation for the initiation rate, $\eta(t)$, in which this function is determined from its past and a delay model results.

We transform the problem into an integral equation by using the solutions of Eqs. S3.2 and S3.4 in the boundary condition and changing variables from $s$ to either $\xi_1$ or $\xi_2$, in regions 1 and 2, respectively. In this form, the integral is evaluated in terms of the value of $\mu z(s,t)$ along the boundary.

As the solution is given in terms of either $z_0(s)$ or $z_1(t)$ according to the region under treatment, different situations arise in obtaining an integral equation. Let $T_L$ and $T_N$ be the times to travel from $s = 0$ at $t = 0$ to $s = L$ and $s = N$, respectively. These are given implicitly in terms of either solution $S_1(\xi_1, \zeta_1)$ or $S_2(\xi_2, \zeta_2)$ as

$$L = S_1(0, T_L) = S_2(0, T_L), \tag{32a}$$

$$N = S_1(0, T_N) = S_2(0, T_N), \tag{32b}$$

see Fig. S1. The three different situations mentioned are: $t < T_L$, $T_L < t < T_N$ and $T_N < t$.

The change of variables required is demonstrated only for $0 < t < T_L$, the other two cases $T_L < t < T_N$ and $T_N < t$ are very similar. The integral from $s = 0$ to $s = L$ is broken in two

$$\int_0^L \mu z(s,t) ds = \int_0^{s_0} \mu z(s,t) ds + \int_{s_0}^L \mu z(s,t) ds, \tag{33}$$

where

$$s_0 = S_1(0,t) = S_2(0,t), \tag{34}$$

is the point where the characteristic dividing the regions intersects the $t$ = constant line. Each integral lies in a different region and so we use solutions of Eqs. S3.2 and S3.4 accordingly.

The integrals are evaluated along $t$ = constant lines, which translates into a relation $\zeta_i = \zeta_i(\xi_i)$, for $i = 1, 2$. In region 1, $\zeta_1(\xi_1) = t$ =constant, the transformation is

$$s = S_1(\xi_1, t). \tag{35}$$

The Jacobian of the transformation, $dS_1/d\xi_1(\xi_1, \zeta_1(\xi_1))$, is obtained by calculating the derivative $\partial_{\xi_1}|_{t=\text{const}}$ of Eq. S3.1b:

$$\frac{\partial}{\partial\zeta_1}\frac{\partial s}{\partial\xi_1}\Big|_{t=\text{const}} = \frac{\partial c_E}{\partial s}\frac{\partial s}{\partial\xi_1}\Big|_{t=\text{const}}. \tag{36}$$

Integrating and using the initial condition $\frac{\partial s}{\partial\xi_1}\Big|_{t=\text{const},\ \zeta_1=0} = 1$, from Eq. S3.1b, the Jacobian is

$$\frac{d}{d\xi_1}S_1(\xi_1, \zeta_1(\xi_1)) = \exp\left\{\int_0^{\zeta_1(\xi_1)} \partial_s c_E(S_1(\xi_1, \zeta_1'), \zeta_1')d\zeta_1'\right\}. \tag{37}$$

For region 2, $\zeta_2(\xi_2) = t - \xi_2$, with $t$ = constant, the transformation is

$$s = S_2(\xi_2, t - \xi_2). \tag{38}$$

Now the Jacobian, $dS_2/d\xi_2(\xi_2, \zeta_2(\xi_2))$, is obtained by calculating the derivative $\partial_{\xi_2}|_{t=\text{const}}$ of Eq. S3.3b:

$$\frac{\partial}{\partial\zeta_2}\frac{\partial s}{\partial\xi_2}\Big|_{t=\text{const}} = \frac{\partial c_E}{\partial s}\frac{\partial s}{\partial\xi_2}\Big|_{t=\text{const}}. \tag{39}$$

The initial condition to this differential equation is found by considering

$$\begin{aligned}\frac{ds}{d\xi_2}\Big|_{t=\text{const}} &= \frac{\partial s}{\partial\xi_2} + \frac{\partial s}{\partial\zeta_2}\frac{d\zeta_2}{d\xi_2}\\ &= \frac{\partial s}{\partial\xi_2} - \frac{\partial s}{\partial\zeta_2}\end{aligned} \tag{40}$$

to evaluate this at $\zeta_2 = 0$, we use the differential equation with its initial condition in Eq. S3.3b. From the first, $\frac{\partial s}{\partial\zeta_2}\Big|_{\zeta_2=0} = c_E(0, \xi_2)$, and from the latter $\frac{\partial s}{\partial\xi_2}\Big|_{\zeta_2=0} = 0$, so that

$$\frac{\partial s}{\partial\xi_2}\Big|_{t=\text{const},\ \zeta_2=0} = -c_E(0, \xi_2). \tag{41}$$

Integrating Eq. S3.12 and using Eq. S3.14, we have

$$\frac{d}{d\xi_2}S_2(\xi_2, \zeta_2(\xi_2)) = -c_E(0, \xi_2) \cdot \exp\left\{\int_0^{\zeta_2(\xi_2)} \partial_s c_E(S_2(\xi_2, \zeta_2'), \zeta_2' + \xi_2)d\zeta_2'\right\}. \tag{42}$$

Changing variables, we obtain

$$\int_0^L \mu z(s,t)ds = \int_0^{s_L(t)} \mu z_0(s')ds' + \int_0^t c_E(0,t')\mu z_1(t')dt' \tag{43}$$

Here for the new limits of integration, it follows from Eq. S3.7 that $s_0$ corresponds to $\xi_1 = \xi_2 = 0$, and from Eqs. S3.3b-S3.3c that $s = 0$ corresponds to $\xi_2 = t$. Here $s_L(t)$ is determined by

$$L = S_1(s_L(t), t). \tag{44}$$

Similarly

$$\int_0^N \mu z(s,t)ds = \int_0^{s_N(t)} \mu z_0(s')ds' + \int_0^t c_E(0,t')\mu z_1(t')dt' \tag{45}$$

and $s_N(t)$ given by

$$N = S_1(s_N(t), t). \tag{46}$$

Similar transformations to the other time intervals yield the desired integral equation for $\eta(t)$.

For $0 < t < T_L$:

$$\eta(t) = \alpha(t)\left(r_T - \int_0^t \eta(t')dt' - \int_0^{s_N(t)} \mu z_0(s')ds'\right) \cdot \left(\mu - \int_0^t \eta(t')dt' - \int_0^{s_L(t)} \mu z_0(s')ds'\right). \tag{47}$$

with $s_L$ and $s_N$ given in Eqs. S3.17 and S3.19.

For $T_L < t < T_N$:

$$\eta(t) = \alpha(t)\left(r_T - \int_0^t \eta(t')dt' - \int_0^{s_N(t)} \mu z_0(s')ds'\right) \cdot \left(\mu - \int_{t_L(t)}^t \eta(t')dt'\right). \tag{48}$$

with $t_L$ given by

$$L = S_2(t_L(t), t - t_L(t)), \tag{49}$$

And finally, for $T_N < t$:

$$\eta(t) = \alpha(t)\left(r_T - \int_{t_N(t)}^t \eta(t')dt'\right) \cdot \left(\mu - \int_{t_L(t)}^t \eta(t')dt'\right). \tag{50}$$

with $t_N(t)$ given by

$$N = S_2(t_N(t), t - t_N(t)), \tag{51}$$

With the concentration of mRNA known, the problem of determining the density $z(s,t)$ has been transformed to that of determining $\eta(t)$ from the integral equation. Then, $z_1(t)$ is obtained from $z_1(t) = \eta(t)/(c_E(0,t)\mu)$ and the ribosome distribution is given by Eq. S3.4.
