# Supplementary material for: The Origins of Time-Delay in Template Biopolymerization Processes
Source: PLoS Comput Biol. 2010 Apr 1;6(4):e1000726. doi: 10.1371/journal.pcbi.1000726 (PMC2848540; doi:10.1371/journal.pcbi.1000726)
Supplement: Text S4 — Equivalence with a Previously Proposed Delay Model (0.03 MB PDF) [file pcbi.1000726.s004.pdf]

Text S4 Equivalence with a Previously Proposed Delay Model

Using our non-dimensional scaling the delay model of [23] has the form

$$\eta(t) = \alpha(r_T - r_B)\left(\mu - \int_{t-\tau_I}^{t} \eta(t')dt'\right), \tag{55a}$$

$$\dot{r}_B = \eta(t) - \eta(t - \tau_E), \tag{55b}$$

applicable when the mRNA chains are empty at $t = 0$, for which $\eta(t) \equiv 0$ for $t < 0$. Further, in that work the delays are defined as

$$\tau_E = \frac{N}{\beta}, \qquad \tau_I = \frac{L}{\beta}. \tag{56}$$

In our PDE-delay model (Text S3), r_B may be given alternatively through the distribution $z(s,t)$ as

$$r_B(t) = \mu \int_0^N z(s,t)ds, \tag{57}$$

or through the initiation rate $\eta(t)$ as

$$r_B(t) = \int_{t_N(t)}^{t} \eta(t')dt', \tag{58}$$

equation S4.4 is obtained from Eq. S4.3 through a change of variables. For the special case of β_j and γ constant and equal to each other, we obtain

$$t_L(t) = t - \tau_I, \tag{59a}$$

$$t_N(t) = t - \tau_N, \tag{59b}$$

and

$$\dot{r}_B(t) = \eta(t) - \eta(t - \tau_E). \tag{60}$$

Using Eqs. S4.4 and S4.5a in 18

$$\eta(t) = \alpha(r_T - r_B)\left(\mu - \int_{t-\tau_I}^{t} \eta(t')dt'\right). \tag{61}$$

Eq. S4.7 is identical to S4.1a and Eq. S4.6 to S4.1b. Therefore, the formulations are completely equivalent, for the special case of α, r_T, and c_E constant. In our PDE formulation, the delays have a slightly different form than Eq. S4.2, as we take made a mean field approximation to account for the quasi-steady state packing in the ribosome fluxes; this has, in fact, the effect of improving the level of approximation.
