# Supplementary material for: The Origins of Time-Delay in Template Biopolymerization Processes
Source: PLoS Comput Biol. 2010 Apr 1;6(4):e1000726. doi: 10.1371/journal.pcbi.1000726 (PMC2848540; doi:10.1371/journal.pcbi.1000726)
Supplement: Text S5 — The Steady State Solution and its Stability (0.04 MB PDF) [file pcbi.1000726.s005.pdf]

Text S5 The Steady State Solution and its Stability

Here we show that when the steady state solution obtained from the PDE model is physically admissible, it is linearly stable. The steady state satisfies

$$\partial_s \left(c_E(s) z_{ss}(s)\right) = 0, \tag{8a}$$

$$c_E(0) z_{ss}(0) = \alpha \left(r_T - \mu \int_0^N z_{ss}(s') ds' \right) \cdot \left(1 - \int_0^L z_{ss}(s') ds' \right), \tag{8b}$$

Eq. S5.1a has the simple solution $z_{ss}(s) = \frac{c_E(0)}{c_E(s)} z_{ss}(0)$, with $z_{ss}(0)$ determined from Eq. S5.1b:

$$z_{ss}(0) = \frac{1}{2\phi\psi} \left[\phi + \psi + \frac{c_E(0)}{\alpha r_T} - \sqrt{\left(\phi + \psi + \frac{c_E(0)}{\alpha r_T}\right)^2 - 4\phi\psi} \right], \tag{9}$$

where $\phi = \frac{\mu}{r_T} \int_0^N \frac{c_E(0)}{c_E(s')} ds'$ and $\psi = \int_0^L \frac{c_E(0)}{c_E(s')} ds'$; the solution is real for all positive values of α, r_T, $c_E(0)$, ϕ and ψ. The minus sign in the quadratic formula must be used in Eq. S5.2 to obtain a physically admissible solution. The inadmissible solution violates the maximum packing of ribosomes, while the solution with the minus sign never violates this physical constraint for all positive values of the parameters.

However, it is still be possible to obtain a physically inadmissible solution that satisfies $\int_0^N z_{ss}(s) ds > N/L$, i.e., more ribosomes on the chain than the number that fit, N/L. This situation arises if the parameters chosen are inconsistent with the approximations performed in the derivation of the PDE model, which does not take ribosome interference into account. For $c_E(s) > 0$, an admissible steady state is always obtained when either μ is big enough, α or r_T are small enough.

We show the steady state solution to be linearly stable, by starting from the integral equation approach and perturbing the steady state initiation rate from equilibrium: $\eta(t) = \eta_{ss} + \tilde{\eta}(t)$, where $\eta_{ss} = \alpha\mu \left(r_T - \mu \int_0^N z_{ss} ds \right) \cdot \left(1 - \int_0^L z_{ss} ds \right)$.

Linearizing, $\tilde{\eta}$ satisfies the equation

$$\tilde{\eta}(t) \simeq -a \int_{t-\tau_L}^{t} \tilde{\eta}(t') dt' - b \int_{t-\tau_N}^{t} \tilde{\eta}(t') dt', \tag{10}$$

where the positive time delays $\tau_I = \int_0^L \frac{ds}{c_E(s)}$ and $\tau_E = \int_0^N \frac{ds}{c_E(s)}$ are the times for the density wave to transverse the initiation site and the complete chain, respectively; $a = \alpha \left(r_T - \eta_{ss} \tau_N \right)$ and $b = \alpha \left(\mu - \eta_{ss} \tau_L \right)$ are both positive constants. The above integral equation may be transformed into a linear delay equation through $\tilde{\eta}(t) = \dot{\sigma}(t)$, and so its characteristic polynomial has an infinite number of eigenvalues [**?**].

With the ansatz $\tilde{\eta}(t) = e^{\lambda t}$, we find that $\lambda = \lambda_r + i\lambda_i$ satisfies

$$1 = -a \frac{1 - e^{-\lambda \tau_L}}{\lambda} - b \frac{1 - e^{-\lambda \tau_N}}{\lambda}. \tag{11}$$

We can rule out the $\lambda = 0$ solution of Eq. S5.4, since in this case the condition $-1 = a\tau_L + b\tau_N$ would need to be fulfilled, with a and b positive. We multiply the whole equation by λ and calculate the

complex modulus $|\lambda|^2$. After rearranging terms, we find

$$\begin{aligned}1 + \frac{a}{2b}(1 - e^{-2\tau_L \lambda_r}) + \frac{b}{2a}(1 - e^{-2\tau_N \lambda_r}) \\ + \left(\frac{1}{a} + \frac{1}{b}\right)\lambda_r + \frac{\lambda_r^2 + \lambda_i^2}{2ab} \\ = e^{-(\tau_L + \tau_N)\lambda_r}\cos[(\tau_N - \tau_L)\,\lambda_i].\end{aligned} \tag{12}$$

For there to exist a solution with $\lambda_r \geq 0$ (excluding $\lambda = 0$) the left hand side of Eq. S5.5 would be necessarily greater than one, while the right hand side is always less than or equal to one. Thus all solutions of Eq. S5.4 must have $\lambda_r < 0$ indicating that η_{ss} is linearly stable.
